# Professor Sérgio Augusto Pereira Novis (1940–2024)

**DOI:** 10.1055/s-0045-1805050

**Published:** 2025-03-19

**Authors:** Luiz Eduardo Novis, Maria Lúcia Vellutini Pimentel, Hélio Afonso Ghizoni Teive

**Affiliations:** 1 Universidade Federal de São Paulo, Escola Paulista de Medicina, Departamento de Neurologia e Neurocirurgia, São Paulo SP, Brazil.; 2 Santa Casa de Misericórdia do Rio de Janeiro, 24 e 25 Enfermaria de Neurologia, Rio de Janeiro RJ, Brazil.; 3 Universidade Federal do Paraná, Hospital de Clínicas, Departamento de Medicina Interna e Serviço de Neurologia, Curitiba PR, Brazil.

**Figure 1:**
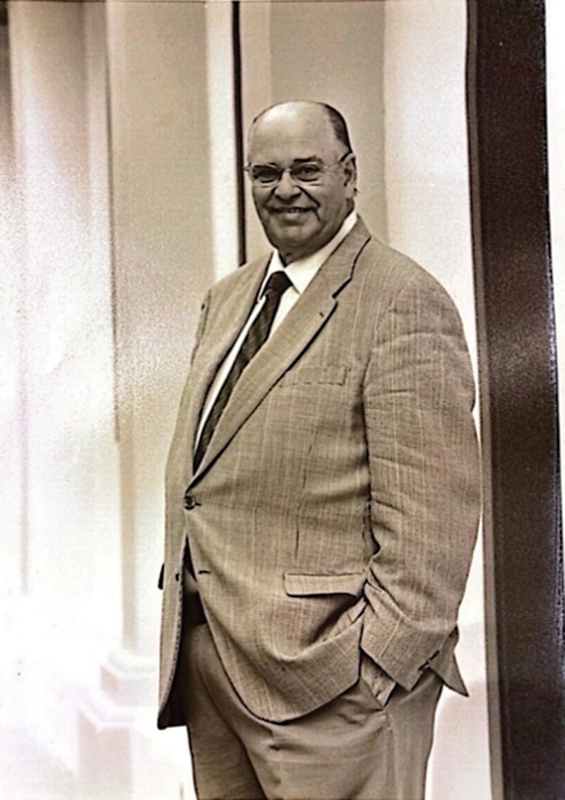
Professor Dr. Sergio Augusto Pereira Novis

Professor Sergio Augusto Pereira Novis was born in the city of Rio de Janeiro on May 16, 1940, to a family that has been dedicated to medicine for six generations. 1 He graduated in Medicine in 1963 from the Rio de Janeiro School of Medicine and Surgery (Escola de Medicina e Cirurgia do Rio de Janeiro, now UNIRIO). He later specialized in Neurology at the Postgraduate Medical School of the Pontifical Catholic University of Rio de Janeiro (Pontifícia Universidade Católica do Rio de Janeiro [PUC-Rio]). In 1967, he became a member of the Brazilian Academy of Neurology (Academia Brasileira de Neurologia [ABN]). 1 In 1971, through a public examination, he obtained the title of full professor ( *livre docente* ) from the Faculty of Medicine of the Federal University of Rio de Janeiro (Universidade Federal do Rio de Janeiro [UFRJ]), defending his thesis entitled *Cerebral astrocytomas: clinical and pathological aspects* . 1 He was elected a full member of the National Academy of Medicine (Academia Nacional de Medicina) in 1987 and was welcomed by Professor Deolindo Couto, at the time Full Professor of Neurology at UFRJ. 1 In 1989, he was elected president of the ABN. Then, in 1993, he was approved in a public competition for the position of Full Professor of Neurology at the Faculty of Medicine of UFRJ, working at the Clementino Fraga Filho University Hospital. 1 2 He was also a Full Professor of Neurology at the Souza Marques School of Medicine, PUC-Rio, and Gama Filho University. 1 In 1997, he became coordinator of the 25 ^th^ and then 24 ^th^ wards (Neurology Service) at Santa Casa do Rio de Janeiro and also worked at the Clínica São Vicente, Rio de Janeiro. 1 In 2011, he became a Professor Emeritus at UFRJ. 1 2 Professor Novis was an emeritus member of the National Academy of Medicine, PUC-Rio, and the National Academy of Rehabilitation Medicine. Professor Sergio Novis was also a full member of the American Academy of Neurology and the Societé Française de Neurologié. 1 2 Professor Sergio Novis became a reference in Brazilian neurology with the creation of a research group in Neurology. His prolific academic production includes several scientific papers, nationally and internationally published in cerebrovascular diseases, multiple sclerosis, as well as neuro-AIDS. 1 2 3 Professor Sergio Novis leaves an important legacy in Brazilian neurology, due to his leadership, entrepreneurship, and brilliant oratory, which will always be remembered by his many students (who even created the Association of Students of Professor Sergio Novis), colleagues, and friends throughout Brazil and the world. 1 2 3 Professor Sergio Novis passed away at the age of 84 on December 12, 2024, in the city of Rio de Janeiro. Professor Novis was an inspired phrase maker. One of the most remarkable is “ *Know what is right and do what is possible* ”.
